# Effect of Tamoxifen on the Risk of Osteoporosis and Osteoporotic Fracture in Younger Breast Cancer Survivors: A Nationwide Study

**DOI:** 10.3389/fonc.2020.00366

**Published:** 2020-03-20

**Authors:** Jihyoun Lee, Heba M. Alqudaihi, Michael Seungcheol Kang, Jisun Kim, Jong Won Lee, Beom Seok Ko, Byung Ho Son, Sei Hyun Ahn, Jong Eun Lee, Sun Wook Han, Zisun Kim, Sung Mo Hur, Ji Sung Lee, Il Yong Chung

**Affiliations:** ^1^Department of Surgery, Soonchunhyang University Seoul Hospital, Seoul, South Korea; ^2^Department of Surgery, Qatif Central Hospital, Ministry of Health, Al Qatif, Saudi Arabia; ^3^Department of Surgery, Asan Medical Center, University of Ulsan College of Medicine, Seoul, South Korea; ^4^Department of Orthopedic Surgery, Asan Medical Center, University of Ulsan College of Medicine, Seoul, South Korea; ^5^Department of Surgery, Soonchunhyang University Cheonan Hospital, Cheonan, South Korea; ^6^Department of Surgery, Soonchunhyang University Bucheon Hospital, Bucheon, South Korea; ^7^Clinical Research Center, Asan Institute for Life Sciences, Asan Medical Center, University of Ulsan College of Medicine, Seoul, South Korea; ^8^Department of Clinical Epidemiology and Biostatistics, Asan Medical Center, University of Ulsan College of Medicine, Seoul, South Korea

**Keywords:** breast neoplasms, survivorship, osteoporosis, bone fractures, tamoxifen

## Abstract

**Background:** Although international guidelines recommend bone screening for premenopausal breast cancer patients taking adjuvant tamoxifen, the effects of tamoxifen on osteoporosis and related risks remain controversial. The objective of this study was to investigate the incidence of and risk factors for osteoporosis and osteoporotic fractures in younger breast cancer patients.

**Methods:** A nationwide retrospective cohort study was conducted using South Korea Health Insurance Review and Assessment Service claims data. The rates of osteoporosis and osteoporotic fracture were calculated as incident cases per person-year and disease-free probability rates were analyzed with the Kaplan-Meier method. To identify risk factors for osteoporosis and osteoporotic fracture, a multivariable Cox proportional hazard regression model was applied.

**Results:** From January 2009 to December 2014, a total of 47,649 breast cancer patients were included. The incidence rates of osteoporosis and osteoporotic fracture were 23.59 and 2.40 per 1,000 person-years, respectively. In the overall population, tamoxifen was significantly associated with a decreased risk of osteoporosis and osteoporotic fractures 0.76). However, tamoxifen was not associated with the risk of osteoporosis (HR 1.24, CI 0.85–1.82) and osteoporotic fracture (HR 8.15, CI 0.36–186.70) in patients under age 40. In the 40–49 years subgroup, tamoxifen significantly decreased the risk of osteoporosis (HR 0.74, CI 0.65–0.84) and osteoporotic fracture (HR 0.49, CI 0.31–0.76).

**Conclusions:** Tamoxifen is not associated with an increased risk of osteoporosis and osteoporotic fracture in premenopausal breast cancer patients. Tailored screening strategies for breast cancer survivors with different osteoporosis risks are needed.

**Precis:** Tamoxifen is not associated with an increased risk of osteoporosis and osteoporotic fracture in premenopausal breast cancer patients. Tailored screening strategies for breast cancer survivors who are at different risks of developing osteoporosis are needed.

## Introduction

As the survival rate of breast cancer patients increases, optimal survivorship care has become an essential part of clinical practice (1, 2). One of the common long-term effects of breast cancer treatments is osteoporosis, with up to 80% of breast cancer patients experiencing bone loss (3, 4). Women with breast cancer, even in the absence of skeletal metastases, are known to have a higher incidence of fractures than women of the same age without breast cancer (5). Aromatase inhibitor (AI) is one of the well-known risk factors for osteoporosis in postmenopausal breast cancer patients (6, 7). Osteoporotic fractures impose an enormous health burden on individuals and take a substantial economic toll on society (8–10).

Tamoxifen is a known risk factor for osteoporosis in premenopausal breast cancer patients. In previous studies involving premenopausal breast cancer patients taking tamoxifen, bone mineral density decreased progressively over a 3-years follow-up period (11), and tamoxifen was associated with significant bone loss in patients who remained premenopausal after adjuvant chemotherapy (12). Based on these studies, the American Cancer Society/American Society of Clinical Oncology (ACS/ASCO) guidelines recommend bone screening every 2 years for premenopausal women receiving tamoxifen (13).

However, these previous studies have limitations. The sample sizes were small and only univariate analyses were performed to calculate the difference between the tamoxifen and placebo groups. Also, the primary outcome was the percent change in bone mineral density (BMD) rather than clinically meaningful outcomes such as osteoporosis or osteoporotic fractures, and the follow-up periods were short. For these reasons, the effect of tamoxifen on osteoporosis risks in premenopausal breast cancer patients remains controversial. Therefore, we conducted this nationwide retrospective cohort study using data from the Health Insurance Review and Assessment Service (HIRA), which archives data from nearly 98% of all citizens in South Korea (14). The objective of this study was to investigate the incidence of and risk factors for osteoporosis and osteoporotic fractures in breast cancer patients and to assess whether tamoxifen is a risk factor for osteoporosis and osteoporotic fractures in younger breast cancer patients.

## Materials and Methods

### Data Source

The HIRA, a governmental organization in South Korea, assesses healthcare services and makes reimbursement decisions under the national healthcare insurance service. The HIRA collects nationwide claims data from healthcare providers (14). The HIRA data consists of six parts: (1) general information; (2) healthcare services; (3) diagnoses; (4) outpatient prescriptions; (5) medication file; and (6) provider information. The diagnostic information is based on the International Classification of Diseases 10th revision (ICD-10).

### Study Population

We selected the study period of January 2007 to December 2017 because of data availability. Newly diagnosed breast cancer was defined by the C50 code (invasive breast cancer) in combination with the specialized V193 claim code, which is an identifier for reimbursement of cancer patients (15). Because we considered a 2-years period before breast cancer diagnosis as a washout period to exclude prevalent breast cancer and any cancer, subjects were excluded from the study if they received a C code within that period. Patients who did not undergo breast cancer surgery and those with a history of *in situ* carcinoma, presumed metastatic breast cancer, preexisting or recent (within 1 year after breast cancer diagnosis) osteoporosis, previous rheumatoid arthritis, or long-term corticosteroid treatment (more than 90 days) were excluded. Male patients or subjects who did not have follow-up claims data after breast cancer diagnosis were also excluded.

**Figure 1:**
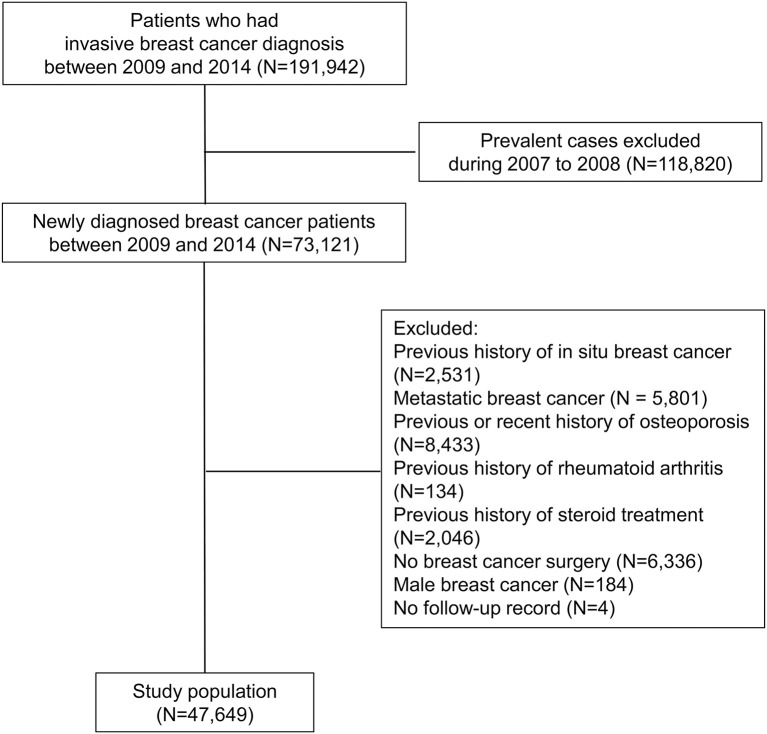
Study population.

From January 2009 to December 2014, a total of 191,942 patients received C50 and V193 codes in the HIRA database. We excluded 118,820 who had *C* codes (any cancer) within the washout period. We excluded 2,531 patients with a previous history of *in situ* carcinoma, 5,801 with metastatic or recurrent breast cancer, 8,433 with preexisting or recently diagnosed osteoporosis, 134 with previous rheumatoid arthritis, 2,046 with long-term corticosteroid treatment, and 6,336 who did not undergo breast cancer surgery. One hundred eighty-seven male patients and 4 patients who did not have follow-up data after breast cancer diagnosis were also excluded (Figure 1).

### Variables and Operational Definitions

Patients' characteristics such as age, type of insurance (health insurance vs. medical aid), and the Charlson Comorbidity Index (CCI) based on ICD-10 codes were analyzed (16). We defined the treatment groups based on claims data within 1 year after breast cancer diagnosis. Radiation therapy (either left and/or right), chemotherapy, ovarian function suppression (OFS), trastuzumab, and endocrine treatment (tamoxifen or AI) were reviewed. Regardless of whether they may have subsequently switched anti-hormonal medications, the patients were allocated into treatment groups according to the initially prescribed endocrine therapy. We defined osteoporosis as the newly claimed osteoporosis codes (M80, M81, M82) in conjunction with at least one of osteoporosis medications (pamidronate, alendronate, ibandronate, risedronate, tibolone, dienogest, estradiol hemihydrate, estradiol valerate, estropipate, conjugated equine estrogens, medroxyprogesterone acetate). The development of osteoporosis was defined using the newly claimed diagnosis in conjunction with medications. Osteoporotic fracture was defined as fracture-related codes (M80, osteoporosis with pathological fracture; S22, fracture of rib, sternum, and thoracic spine; S32, fracture of lumbar spine and pelvis; S52, fracture of forearm; S62, fracture at wrist and hand; S72, fracture of femur) or treatment of fractures and osteoporosis within 6 months before or after the fracture.

### Statistical Analysis

The baseline characteristics of the included patients are presented as the number of patients (%) or mean ± SD. The incidence rates of osteoporosis and osteoporotic fracture were calculated by dividing the number of incident cases by the total follow-up period (person-years). The disease-free probability of osteoporosis and osteoporotic fracture were calculated by the Kaplan-Meier method, and the log-rank test was performed to confirm differences across risk factors.

For the identification of risk factors for osteoporosis and osteoporotic fracture, a multivariable Cox proportional hazard regression model was applied and adjusted hazard ratio (HR) and 95% confidence interval (CI) were estimated. Age, type of insurance, CCI, chemotherapy, endocrine therapy, OFS, radiotherapy, and trastuzumab were selected as covariates for regression models. Subgroup analyses were performed within age groups (<40, 40–49, 50–59, 60–69, and ≥70 years) to further clarify risk factors of osteoporosis and osteoporotic fracture.

Statistical analyses were performed with SAS software (version 9.4, SAS Institute, Cary, NC, USA). This study was approved by the Soonchunhyang University Seoul Hospital Institutional Review Board (IRB no. SCHUH 2018-11-011).

## Results

### Baseline Characteristics

A total of 47,649 breast cancer survivors were included in this analysis. Among them, the proportion aged 40–49 years at the time of diagnosis was 42.04 % (Table 1). The proportion of breast cancer survivors who received any type of chemotherapy was 67.57% (*n* = 32,198). More than two-thirds of the survivors received endocrine treatment, and tamoxifen was the most frequently prescribed agent. OFS with goserelin or leuprolide was prescribed in 10.54% of the survivors. All the subjects underwent breast cancer surgeries, according to our operational definition.

**Table 1:** Baseline characteristics and adjuvant treatments in breast cancer survivors.

	**Number (%)**
**Total**	47,649
Age at diagnosis^*^	48.92 ± 9.82
<30	657 (1.38)
30–39	6,464 (13.57)
40–49	20,034 (42.04)
50–59	13,968 (29.31)
60–69	4,808 (10.09)
70–79	1,510 (3.17)
80-	208 (0.44)
Charlson comorbidity index^*^	1.34 ± 1.42
0	15,579 (32.70)
1	15,081 (31.65)
2	9,120 (19.14)
3	4,179 (8.77)
4	1,891 (3.97)
≥5	1,799 (3.76)
**Reimbursement type**	
National health insurance	46,871 (98.37)
Medical aid	777 (1.63)
Others	1 (0.00)
**Chemotherapy(any)**	
No	15,451 (32.43)
Yes	32,198 (67.57)
**Endocrine treatment**	
No	13,312 (27.94)
Tamoxifen	24,006 (50.38)
Aromatase inhibitor^**^	10,331 (21.68)
Ovarian function suppression^***^	
No	42,627 (89.46)
Yes	5,022 (10.54)
**Radiotherapy**	
No	13,301 (27.91)
Yes	34,348 (72.09)
**Trastuzumab**	
No	40,930 (85.90)
Yes	6,719 (14.10)

* presented as Mean ± SD, measured at breast cancer diagnosis.

** letrozole, anastrozole, exemestane.

*** *goserelin, leuprolide*.

### Incidence of Osteoporosis and Osteoporotic Fracture

During the study period, 5,955 osteoporosis events were observed in 252,396 person-years. The incidence rate of osteoporosis in breast cancer survivors was 23.59 per 1000 person-years (95% CI, 23.00–24.20). Osteoporotic fracture incidence was assessed as 2.40 per 1000 person-years (95% CI, 2.23–2.60), with 647 events occurring in 269,075 person-years (Table 2). Age at diagnosis and CCI were significantly associated with development of osteoporosis (*p* < 0.0001) and osteoporotic fracture (*p* < 0.0001) in the univariate analysis. The incidence rate of osteoporosis was highest in patients aged 70–79 years. The risk of osteoporotic fracture was highest in patients older than 80 years (17.16 per 1,000 person-years) followed by patients aged 70–79 years. Event-free probability of osteoporosis and osteoporotic fracture after 1 year following breast cancer diagnosis is presented in Supplementary Figure 1.

**Table 2:** Incidence rates of osteoporosis and osteoporotic fracture according to age at diagnosis and comorbidities.

	**N**	**Events** **(*n*)**	**Total person-year**	**Incidence rate** **(per 1,000 person-year)**	**HR** **(95% CI)**	p^*^
**Osteoporosis**						
**Total**	47,649	5,955	2,52,396	23.59 (23.00–24.20)		
**Age at diagnosis**					<0.0001	
<30	657	6	3,771	1.59 (0.71–3.54)	1 (Ref)	
30–39	6,464	161	37,017	4.35 (3.73–5.08)	2.73 (1.21–6.16)	
40–49	20,034	1521	1,10,010	13.83 (13.15–14.54)	8.67 (3.89–19.34)	
50–59	13,968	2,409	71,240	33.82 (32.49–35.19)	21.33 (9.57–47.52)	
60–69	4,808	1,360	22,766	59.74 (56.65–63.00)	37.97 (17.03–84.65)	
70–79	1,510	455	6,773	67.18 (61.28–73.64)	42.98 (19.21–96.18)	
80+	208	43	819	52.50 (38.94–70.79)	33.82 (14.39–79.45)	
CCI^**^						<0.0001
0	15,579	1,586	88,502	17.92 (17.06–18.82)	1 (Ref)	
1	15,081	1,799	80,528	22.34 (21.33–23.40)	1.24 (1.16–1.33)	
2	9,120	1,225	45,839	26.72 (25.27–28.26)	1.48 (1.38–1.60)	
3	4,179	682	20,203	33.76 (31.32–36.39)	1.88 (1.72–2.05)	
4	1,891	334	9,079	36.79 (33.05–40.95)	2.05 (1.82–2.30)	
≥5	1,799	329	8,245	39.90 (35.81–44.45)	2.22 (1.97–2.50)	
**Osteoporotic fracture**						
**Total**	47,649	647	2,69,075	2.40 (2.23–2.60)		<0.0001
**Age at diagnosis**						
<40	7,121	9	41,247	0.22 (0.11–0.42)	1 (Ref)	
40–49	20,034	110	1,14,136	0.96 (0.80–1.16)	4.45 (2.25–8.77)	
50–59	13,968	221	78,053	2.83 (2.48–3.23)	13.15 (6.75–25.61)	
60–69	4,808	180	26,762	6.73 (5.81–7.78)	31.20 (15.97–60.94)	
70–79	1,510	111	7,944	13.97 (11.60–16.83)	65.87 (33.40–129.94)	
80+	208	16	933	17.16 (10.51–28.00)	85.30 (37.69–193.08)	
CCI^**^						<0.0001
0	15,579	156	93,225	1.67 (1.43–1.96)	1 (Ref)	
1	15,081	178	85,669	2.08 (1.79–2.41)	1.27 (1.03–1.58)	
2	9,120	129	49,196	2.62 (2.21–3.12)	1.64 (1.30–2.08)	
3	4,179	83	22,033	3.77 (3.04–4.67)	2.38 (1.82–3.11)	
4	1,891	50	9,918	5.04 (3.82–6.65)	3.19 (2.32–4.39)	
≥5	1,799	51	9,034	5.65 (4.29–7.43)	3.63 (2.64–4.98)	

* p-value from log-rank test.

** *CCI, Charlson Comorbidity Index*.

### Risk Factors for Osteoporosis and Osteoporotic Fracture According to Age at Diagnosis

Factors associated with the incidence of osteoporosis and osteoporotic fracture showed different patterns according to age subgroups (Table 3). In patients younger than 40 years, the use of OFS was significantly related to an increased incidence of osteoporosis. In the age 40–49 group, chemotherapy, AI and OFS were significantly associated with an increased risk of osteoporosis. In patients aged 50–69 years, AI significantly increased the risk of osteoporosis; in contrast, tamoxifen was associated with a decreased risk of osteoporosis.

**Table 3:** Factors associated with osteoporosis according to age subgroups in univariate and multivariate analysis.

	**N**	**Events (*n*)**	**Total person-year**	**Incidence rate** **(per 1,000 person-year)**	p^*^	**Crude HR** **(95% CI)**	**Adjusted HR** **(95% CI)**
**Age <40**							
**Reimbursement type**					0.1178		
National health insurance	7,068	164	40,477	4.05 (3.48–4.72)		1 (Ref)	1 (Ref)
Medical aid	53	3	311	9.66 (3.11–29.94)		2.42 (0.77–7.57)	2.49 (0.85–7.25)
**Chemotherapy(any)**					0.5516		
No	1,878	41	10,837	3.78 (2.79–5.14)		1 (Ref)	1 (Ref)
Yes	5,243	126	29,951	4.21 (3.53–5.01)		1.11 (0.78–1.58)	1.39 (0.95–2.02)
**Endocrine treatment**					0.0363		
No	2,364	40	13,446	2.97 (2.18–4.06)		1 (Ref)	1 (Ref)
Tamoxifen	4,736	127	27,220	4.67 (3.92–5.55)		1.55 (1.08–2.21)	1.24 (0.85–1.82)
Aromatase inhibitor^**^	21	0	121	0.00		1.34 (0.08–22.02)	1.33 (0.08–22.34)
Ovarian function suppression^***^					<0.0001		
No	5,632	108	32,433	3.33 (2.76–4.02)		1 (Ref)	1 (Ref)
Yes	1,489	59	8,355	7.06 (5.47–9.11)		2.12 (1.54–2.91)	2.15 (1.52–3.06)
**Radiotherapy**					0.859		
No	1,864	45	10,761	4.18 (3.12–5.60)		1 (Ref)	1 (Ref)
Yes	5,257	122	30,027	4.06 (3.40–4.85)		0.97 (0.69–1.36)	0.92 (0.65–1.30)
**Trastuzumab**					0.8198		
No	6,119	146	35,440	4.12 (3.50–4.85)		1 (Ref)	1 (Ref)
Yes	1,002	21	5,348	3.93 (2.56–6.02)		0.95 (0.60–1.50)	0.99 (0.62–1.59)
**Age 40–49**							
**Reimbursement type**					0.7624		
National health insurance	19,729	1,499	1,08,318	13.84 (13.16–14.56)		1 (Ref)	1 (Ref)
Medical aid	305	22	1,693	13.00 (8.56–19.74)		0.94 (0.62–1.43)	0.81 (0.53–1.24)
**Chemotherapy(any)**					<0.0001		
No	6,424	381	35,732	10.66 (9.64–11.79)		1 (Ref)	1 (Ref)
Yes	13,610	1,140	74,278	15.35 (14.48–16.27)		1.45 (1.29–1.62)	1.41 (1.24–1.60)
**Endocrine treatment**					<0.0001		
No	4,448	383	23,888	16.03 (14.51–17.72)		1 (Ref)	1 (Ref)
Tamoxifen	14,710	950	81,377	11.67 (10.95–12.44)		0.72 (0.64–0.82)	0.74 (0.65–0.84)
Aromatase inhibitor^**^	876	188	4,745	39.62 (34.34–45.71)		2.47 (2.08–2.94)	2.45 (2.05–2.92)
Ovarian function suppression^***^					0.0113		
No	16,838	1,314	92,422	14.22 (13.47–15.01)		1 (Ref)	1 (Ref)
Yes	3,196	207	17,588	11.77 (10.27–13.49)		0.83 (0.71–0.96)	1.19 (1.01–1.40)
**Radiotherapy**					0.0548		
No	5,130	427	28,435	15.02 (13.66–16.51)		1 (Ref)	1 (Ref)
Yes	14,904	1,094	81,575	13.41 (12.64–14.23)		0.90 (0.80–1.00)	0.87 (0.77–0.97)
**Trastuzumab**					0.0282		
No	17,546	1,320	97,261	13.57 (12.86–14.32)		1 (Ref)	1 (Ref)
Yes	2,488	201	12,749	15.77 (13.73–18.10)		1.18 (1.02–1.37)	1.04 (0.89–1.21)
**Age 50–59**							
**Reimbursement type**					0.9962		
National health insurance	13,763	2,374	70,202	33.82 (32.48–35.20)		1 (Ref)	1 (Ref)
Medical aid	205	35	1,038	33.73 (24.22–46.98)		1.00 (0.72–1.40)	1.01 (0.72–1.41)
**Chemotherapy(any)**					<0.0001		
No	4,166	643	21,789	29.51 (27.32–31.88)		1 (Ref)	1 (Ref)
Yes	9,802	1,766	49,452	35.71 (34.08–37.42)		1.21 (1.11–1.33)	1.09 (0.99–1.20)
**Endocrine treatment**					<0.0001		
No	4,526	752	22,869	32.88 (30.61–35.32)		1 (Ref)	1 (Ref)
Tamoxifen	3,555	405	19,237	21.05 (19.10–23.21)		0.64 (0.56–0.72)	0.69 (0.61–0.78)
Aromatase inhibitor^**^	5,887	1,252	29,134	42.97 (40.66–45.42)		1.31 (1.19–1.43)	1.34 (1.22–1.47)
Ovarian function suppression^***^					<0.0001		
No	13,631	2,385	69,368	34.38 (33.03–35.79)		1 (Ref)	1 (Ref)
Yes	337	24	1,872	12.82 (8.59–19.13)		0.37 (0.25–0.56)	0.61 (0.40–0.92)
**Radiotherapy**					0.8216		
No	3,732	657	19,327	33.99 (31.49–36.69)		1 (Ref)	1 (Ref)
Yes	10,236	1,752	51,913	33.75 (32.21–35.37)		0.99 (0.90–1.08)	0.94 (0.86–1.03)
**Trastuzumab**					0.0158		
No	11,535	1,970	59,582	33.06 (31.64–34.56)		1 (Ref)	1 (Ref)
Yes	2,433	439	11,658	37.66 (34.29–41.35)		1.14 (1.02–1.26)	1.09 (0.98–1.22)
**Age 60–69**							
**Reimbursement type**					0.0231		
National health insurance	4,673	1,311	22,166	59.15 (56.03–62.44)		1 (Ref)	1 (Ref)
Medical aid	135	49	600	81.65 (61.71–108.03)		1.39 (1.05–1.85)	1.42 (1.06–1.89)
**Chemotherapy(any)**					0.0450		
No	1,778	483	8,679	55.65 (50.91–60.85)		1 (Ref)	1 (Ref)
Yes	3,030	877	14,087	62.25 (58.27–66.51)		1.12 (1.00–1.25)	1.11 (0.99–1.25)
**Endocrine treatment**					<0.0001		
No	1,463	389	6,818	57.05 (51.66–63.01)		1 (Ref)	1 (Ref)
Tamoxifen	651	151	3,357	44.98 (38.35–52.76)		0.79 (0.65–0.95)	0.81 (0.67–0.99)
Aromatase inhibitor^**^	2,694	820	12,591	65.13 (60.82–69.74)		1.14 (1.01–1.28)	1.18 (1.04–1.34)
Ovarian function suppression^***^					NA		
No	4,808	1,360	22,766	59.74 (56.65–63.00)	NA	NA	NA
Yes	0	NA	NA	NA	NA	NA	NA
**Radiotherapy**					0.3174		
No	1,584	469	7,597	61.73 (56.39–67.58)		1 (Ref)	1 (Ref)
Yes	3,224	891	15,168	58.74 (55.01–62.73)		0.94 (0.84–1.06)	0.92 (0.83–1.04)
**Trastuzumab**					0.7159		
No	4,121	1,170	19,683	59.44 (56.13–62.95)		1 (Ref)	1 (Ref)
Yes	687	190	3,082	61.64 (53.47–71.06)		1.03 (0.88–1.20)	1.01 (0.86–1.19)
**Age** **≥70**							
**Reimbursement type**					0.665		
National health insurance	1,638	476	7,225	65.88 (60.22–72.08)		1 (Ref)	1 (Ref)
Medical aid	80	22	367	59.90 (39.44–90.96)		0.91 (0.59–1.40)	0.91 (0.59–1.39)
**Chemotherapy(any)**					0.0289		
No	1,205	333	5,416	61.49 (55.22–68.46)		1 (Ref)	1 (Ref)
Yes	513	165	2,176	75.81 (65.08–88.31)		1.23 (1.02–1.48)	1.18 (0.96–1.45)
**Endocrine treatment**					0.0126		
No	511	139	2,132	65.19 (55.21–76.98)		1 (Ref)	1 (Ref)
Tamoxifen	354	86	1,713	50.22 (40.65–62.03)		0.77 (0.59–1.01)	0.83 (0.63–1.10)
Aromatase inhibitor^**^	853	273	3,747	72.85 (64.70–82.02)		1.11 (0.91–1.36)	1.19 (0.96–1.47)
Ovarian function suppression^***^					NA		
No	1,718	498	7,592	65.59 (60.08–71.61)	NA	NA	NA
Yes	0	NA	NA	NA	NA	NA	NA
**Radiotherapy**					0.3657		
No	991	277	4,399	62.97 (55.97–70.84)		1 (Ref)	1 (Ref)
Yes	727	221	3,193	69.21 (60.66–78.97)		1.09 (0.91–1.30)	1.05 (0.87–1.25)
**Trastuzumab**					0.0760		
No	1,609	460	7,157	64.27 (58.66–70.42)		1 (Ref)	1 (Ref)
Yes	109	38	435	87.33 (63.55–120.02)		1.35 (0.97–1.88)	1.22 (0.85–1.75)

NA, not available.

* log-rank test.

** letrozole, anastrozole, exemestane.

*** *goserelin, leuprolide*.

There were only 9 cases of osteoporotic fracture in 7,121 patients younger than 40 years, and OFS was not associated with an increased risk of osteoporotic fracture in these patients (Supplementary Table 1). An increased risk of osteoporotic fracture was significantly associated with chemotherapy (HR, 1.75; 95% CI, 1.07–2.88) and AI (HR, 2.35; 95% CI, 1.34–4.12) in the age 40–49 subgroup.

### Effect of Tamoxifen on Bone Health in Younger Breast Cancer Patients

**Figure 2:**
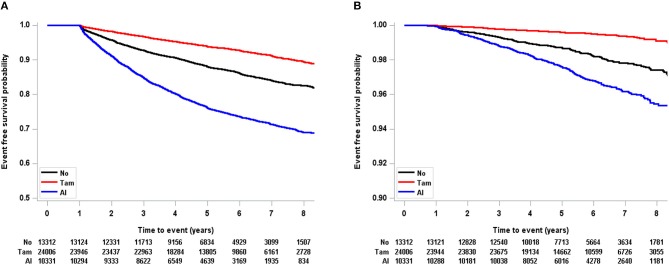
Kaplan-Meier event free probability according to endocrine treatment in total population (**A**: osteoporosis, **B**: osteoporotic fracture). Tam, tamoxifen; AI, aromatase inhibitor.

In the total population, tamoxifen was significantly associated with a decreased risk of osteoporosis and osteoporotic fracture (Figure 2). The risk of osteoporosis (HR, 1.24; CI, 0.85–1.82) and osteoporotic fracture (HR, 8.15; CI, 0.36–186.70) was not associated with tamoxifen in patients younger than 40 years (Table 3 and Supplementary Table 1). However, in the age 40–49 group, tamoxifen significantly decreased the risk of osteoporosis (HR, 0.74; CI, 0.65–0.84) and osteoporotic fracture (HR, 0.49; CI, 0.31–0.76).

## Discussion

The results of this large retrospective study show that the use of adjuvant tamoxifen does not increase the risk of osteoporosis and osteoporotic fracture in younger breast cancer survivors. In patients younger than 40 years at the time of breast cancer diagnosis, adjuvant tamoxifen was not associated with the development of osteoporosis and osteoporotic fracture. Furthermore, tamoxifen significantly decreased the risk of osteoporosis and osteoporotic fracture in breast cancer patients aged 40–49 years at the time of diagnosis. OFS was significantly associated with an increased risk of osteoporosis in patients younger than 50 years. Chemotherapy and AI were significantly related to the risk of osteoporosis and osteoporotic fracture in patients between the ages of 40 and 49 years.

The results of this study differ from those of previous studies. Previous studies showed an association between tamoxifen use and bone loss in premenopausal patients. One previous study showed an annual loss of BMD of 1.44% in premenopausal breast cancer patients on tamoxifen (11). However, only 125 premenopausal breast cancer patients were enrolled in that study and treatment variables that can influence BMD were not statistically adjusted. Another study showed a 4.6% decrease in BMD at the 3-years follow-up evaluation in premenopausal patients taking tamoxifen after adjuvant chemotherapy. The number of patients enrolled in that study was also small, and only univariate analyses were conducted (12). Recently, researchers from Germany reported that tamoxifen increased the risk of fracture in premenopausal breast cancer patients compared to control patients without cancer (17). However, the selection criteria for the non-cancer control patients were not able to demonstrate the effect of tamoxifen on fracture because adjuvant treatments such as OFS and chemotherapy could not be statistically adjusted. To address these drawbacks, we conducted this nationwide retrospective cohort study.

To our knowledge, this is the largest retrospective cohort study of the effect of tamoxifen on bone health in younger breast cancer patients. Approximately 27,000 breast cancer patients younger than 50 years were enrolled, comprising 150,798 person-years. The results of this study indicate that tamoxifen does not increase the risk of osteoporosis and osteoporotic fracture in younger breast cancer survivors. These results contradict the ASCO recommendation that primary clinicians should refer premenopausal breast cancer survivors who are taking tamoxifen for repeat bone screening every 2 years (13).

One of the interesting findings of this study is that tamoxifen significantly decreased the risk of osteoporosis and osteoporotic fracture in breast cancer patients aged 40 to 49 years at the time of diagnosis. This can be explained by their perimenopausal status and long-term treatment with adjuvant tamoxifen. For patients with hormone receptor-positive breast cancers and lymph node metastasis, 10 years of adjuvant tamoxifen treatment are usually recommended (18). With the extended tamoxifen therapy, premenopausal women who are premenopausal at the time of breast cancer diagnosis might continue to take tamoxifen beyond the start of menopause, after which tamoxifen would show a protective effect on bone health.

In postmenopausal patients, AI increases the incidence of osteoporosis and osteoporotic fracture. The current study, in accordance with other reports (19, 20), demonstrated a marked increase in the risk of osteoporosis with AI, reflecting the near-complete estrogen depletion and subsequent disruption in bone homeostasis caused by these agents (21).

Multiple factors are related to the incidence of osteoporosis. The most common causes of bone loss in women are menopause and aging. Aging is associated with greater bone resorption and less bone formation, whereas menopause induces accelerated bone loss due to lowering levels of endogenous estrogen (22). In HIRA data study about the burden of osteoporosis in the general population, the prevalence of osteoporosis increased with age; the peak was at 70–79 years, with a rate of 5,253 diagnoses per 10,000 persons (23). Although we cannot directly compare this to the results of our study because of the different operational definitions, we similarly found an increasing incidence of osteoporosis in the older age group.

The limitations of this study should be noted. First, we were not able to perform survival analysis because the HIRA data is claims-based in accordance with the Personal Information Protection Act in Korea. Therefore, we could not assess the effect of osteoporosis and osteoporotic fracture on overall survival. Second, endocrine therapy treatment group allocation was based on claims data from the 1st year after diagnosis, and some patients may have subsequently switched from tamoxifen to AI. Third, as we defined osteoporosis as osteoporosis diagnosis codes in combination with osteoporosis medications, osteoporosis patients to whom osteoporosis medications were not prescribed due to other medical conditions were not included in this analysis. Lastly, due to the limitations of claims data, we were unable to gather information about diet, exercise, exposure to sunlight, and vitamin D supplementation, which are important factors for maintaining bone health.

The results of this study should be interpreted in the context of the study period. First, we did not analyze the use of denosumab which has been reimbursed for osteoporosis treatment from 2018 in South Korea. The resulting change in clinical practice could potentially affect the study outcomes. Second, after the practice-changing report from the Suppression of Ovarian Function Trial in 2015, adjuvant ovarian suppression in premenopausal breast cancer patients who remain premenopausal after chemotherapy, especially young patients, is recommended (24). Although OFS was not associated with a significantly increased risk of osteoporotic fracture in patients younger than 40 years in this study, OFS is now more often prescribed, possibly affecting the incidence of osteoporosis and osteoporotic fractures.

In conclusion, tamoxifen is not associated with an increased risk of osteoporosis and osteoporotic fracture in premenopausal breast cancer patients. Risk factors for osteoporosis and osteoporotic fractures vary according to patient age. Tailored screening strategies for breast cancer survivors who are at different risks of developing osteoporosis are needed.

## Data Availability Statement

The datasets generated for this study will not be made publicly available. The datasets are from the Korean National database which is not allowed to be extracted from the server.

## Ethics Statement

The studies involving human participants were reviewed and approved by Soonchunhyang University Seoul Hospital Institutional Review Board (2018-11-011). Written informed consent from the participants' legal guardian/next of kin was not required to participate in this study in accordance with the national legislation and the institutional requirements.

## Author Contributions

JLe and HA: planned, wrote and revised the article. JK, MK, JWL, BK, BS, SA, JEL, SHa, ZK, and SHu: reviewed and edited the article. JSL: planned, analyzed data and statistics. IC: planned, wrote, revised, and supervised the concept of the article.

### Conflict of Interest

The authors declare that the research was conducted in the absence of any commercial or financial relationships that could be construed as a potential conflict of interest. Reviewer MI is currently organizing a Research Topic with one of the authors JK, and confirms the absence of any other collaboration.

## Supplementary Material

Supplementary Figure 1 Kaplan-Meier event free probability in total population (**A**: osteoporosis, **B**: osteoporotic fracture).

Supplementary Table 1 Factors associated with osteoporotic fracture according to age subgroups in univariate and multivariate analysis.
