# Supplementary figures and images for: Effect of Tamoxifen on the Risk of Osteoporosis and Osteoporotic Fracture in Younger Breast Cancer Survivors: A Nationwide Study
Source: Front Oncol. 2020 Mar 20;10:366. doi: 10.3389/fonc.2020.00366 (PMC7098996; doi:10.3389/fonc.2020.00366)

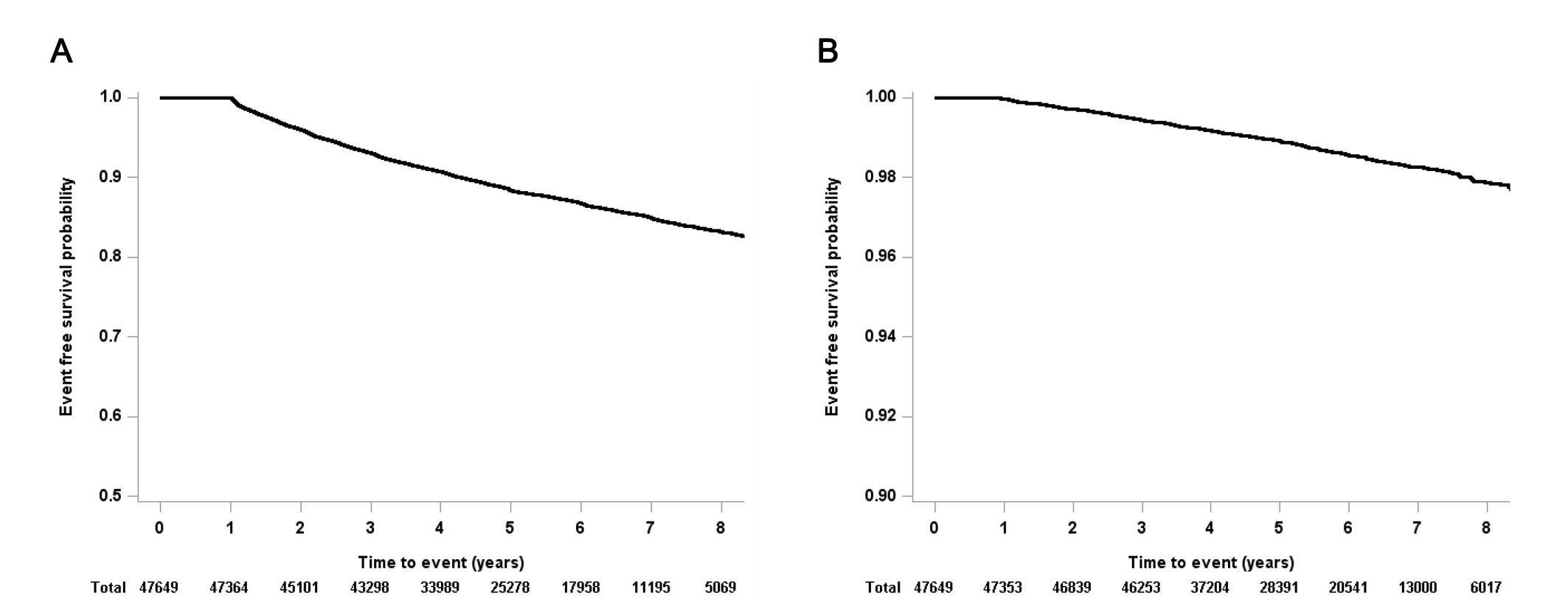

Supplement: Supplementary Figure 1 — Kaplan-Meier event free probability in total population (A: osteoporosis, B: osteoporotic fracture). [file Image_1.TIF]
