# Supplementary material for: Effect of Tamoxifen on the Risk of Osteoporosis and Osteoporotic Fracture in Younger Breast Cancer Survivors: A Nationwide Study
Source: Front Oncol. 2020 Mar 20;10:366. doi: 10.3389/fonc.2020.00366 (PMC7098996; doi:10.3389/fonc.2020.00366)
Supplement: Supplementary Table 1 — Factors associated with osteoporotic fracture according to age subgroups in univariate and multivariate analysis. [file Table_1.DOCX]

**Supplementary Table 1.** **Factors associated with osteoporotic fracture according to age subgroups in univariate and multivariate analysis**

|  | **N** | **Events**  **(n)** | **Total person-year** | **Incidence rate**  **(per 1000 person-year)** | ***p*^a^** | **Crude HR**  **(95% CI)** | **Adjusted HR**  **(95% CI)** |
| --- | --- | --- | --- | --- | --- | --- | --- |
| **Age<40** |  |  |  |  |  |  |  |
| **Reimbursement type** |  |  |  |  | 0.7892 |  |  |
| National health insurance | 7068 | 9 | 40928 | 0.22 (0.11-0.42) |  | 1 (Ref) | 1 (Ref) |
| Medical aid | 53 | 0 | 319 | 0.00 |  | 6.62 (0.33-133.72) | 4.17 (0.22-80.42) |
| **Chemotherapy(any)** |  |  |  |  | 0.6575 |  |  |
| No | 1878 | 3 | 10974 | 0.27 (0.09-0.85) |  | 1 (Ref) | 1 (Ref) |
| Yes | 5243 | 6 | 30273 | 0.20 (0.09-0.44) |  | 0.68 (0.17-2.67) | 1.20 (0.28-5.17) |
| **Endocrine treatment** |  |  |  |  | 0.1072 |  |  |
| No | 2364 | 0 | 13563 | 0.00 |  | 1 (Ref) | 1 (Ref) |
| Tamoxifen | 4736 | 9 | 27563 | 0.33 (0.17-0.63) |  | 9.35 (0.43-201.87) | 8.15 (0.36-186.70) |
| Aromatase inhibitor^b^ | 21 | 0 | 121 | 0.00 |  | 113.63 (1.65-7848.03) | 78.83 (0.88-7072.36) |
| **Ovarian function suppression^c^** | |  |  |  | 0.0787 |  |  |
| No | 5632 | 5 | 32716 | 0.15 (0.06-0.37) |  | 1 (Ref) | 1 (Ref) |
| Yes | 1489 | 4 | 8531 | 0.47 (0.18-1.25) |  | 3.14 (0.84-11.66) | 1.82 (0.44-7.47) |
| **Radiotherapy** |  |  |  |  | 0.2214 |  |  |
| No | 1864 | 4 | 10901 | 0.37 (0.14-0.98) |  | 1 (Ref) | 1 (Ref) |
| Yes | 5257 | 5 | 30346 | 0.16 (0.07-0.40) |  | 0.44 (0.12-1.64) | 0.43 (0.11-1.64) |
| **Trastuzumab** |  |  |  |  | 0.2429 |  |  |
| No | 6119 | 9 | 35848 | 0.25 (0.13-0.48) |  | 1 (Ref) | 1 (Ref) |
| Yes | 1002 | 0 | 5400 | 0.00 |  | 0.35 (0.02-6.98) | 0.40 (0.02-9.35) |
| **Age 40-49** |  |  |  |  |  |  |  |
| **Reimbursement type** |  |  |  |  | 0.3164 |  |  |
| National health insurance | 19729 | 107 | 112385 | 0.95 (0.79-1.15) |  | 1 (Ref) | 1 (Ref) |
| Medical aid | 305 | 3 | 1751 | 1.71 (0.55-5.31) |  | 2.07 (0.71-6.04) | 1.54 (0.53-4.48) |
| **Chemotherapy(any)** |  |  |  |  | 0.0096 |  |  |
| No | 6424 | 23 | 36759 | 0.63 (0.42-0.94) |  | 1 (Ref) | 1 (Ref) |
| Yes | 13610 | 87 | 77377 | 1.12 (0.91-1.39) |  | 1.79 (1.13-2.83) | 1.75 (1.07-2.88) |
| **Endocrine treatment** |  |  |  |  | <.0001 |  |  |
| No | 4448 | 36 | 24897 | 1.45 (1.04-2.00) |  | 1 (Ref) | 1 (Ref) |
| Tamoxifen | 14710 | 55 | 83910 | 0.66 (0.50-0.85) |  | 0.45 (0.30-0.69) | 0.49 (0.31-0.76) |
| Aromatase inhibitor^b^ | 876 | 19 | 5329 | 3.57 (2.27-5.59) |  | 2.39 (1.37-4.16) | 2.35 (1.34-4.12) |
| **Ovarian function suppression^c^** | |  |  |  | 0.0522 |  |  |
| No | 16838 | 100 | 95957 | 1.04 (0.86-1.27) |  | 1 (Ref) | 1 (Ref) |
| Yes | 3196 | 10 | 18180 | 0.55 (0.30-1.02) |  | 0.55 (0.29-1.05) | 1.07 (0.53-2.18) |
| **Radiotherapy** |  |  |  |  | <.0001 |  |  |
| No | 5130 | 47 | 29576 | 1.59 (1.19-2.12) |  | 1 (Ref) | 1 (Ref) |
| Yes | 14904 | 63 | 84561 | 0.75 (0.58-0.95) |  | 0.48 (0.33-0.70) | 0.46 (0.32-0.68) |
| **Trastuzumab** |  |  |  |  | 0.7584 |  |  |
| No | 17546 | 99 | 100845 | 0.98 (0.81-1.20) |  | 1 (Ref) | 1 (Ref) |
| Yes | 2488 | 11 | 13292 | 0.83 (0.46-1.49) |  | 0.94 (0.51-1.74) | 0.71 (0.38-1.34) |
| **Age 50-59** |  |  |  |  |  |  |  |
| **Reimbursement type** |  |  |  |  | 0.6575 |  |  |
| National health insurance | 13763 | 217 | 76926 | 2.82 (2.47-3.22) |  | 1 (Ref) | 1 (Ref) |
| Medical aid | 205 | 4 | 1127 | 3.55 (1.33-9.46) |  | 1.41 (0.55-3.58) | 1.48 (0.58-3.79) |
| **Chemotherapy(any)** |  |  |  |  | 0.0120 |  |  |
| No | 4166 | 50 | 23583 | 2.12 (1.61-2.80) |  | 1 (Ref) | 1 (Ref) |
| Yes | 9802 | 171 | 54470 | 3.14 (2.70-3.65) |  | 1.48 (1.08-2.03) | 1.25 (0.90-1.73) |
| **Endocrine treatment** |  |  |  |  | <.0001 |  |  |
| No | 4526 | 86 | 24922 | 3.45 (2.79-4.26) |  | 1 (Ref) | 1 (Ref) |
| Tamoxifen | 3555 | 24 | 20312 | 1.18 (0.79-1.76) |  | 0.34 (0.22-0.54) | 0.37 (0.23-0.60) |
| Aromatase inhibitor^b^ | 5887 | 111 | 32819 | 3.38 (2.81-4.07) |  | 0.98 (0.74-1.30) | 0.98 (0.73-1.31) |
| **Ovarian function suppression^c^** | |  |  |  | 0.0501 |  |  |
| No | 13631 | 220 | 76110 | 2.89 (2.53-3.30) |  | 1 (Ref) | 1 (Ref) |
| Yes | 337 | 1 | 1943 | 0.51 (0.07-3.65) |  | 0.26 (0.05-1.32) | 0.66 (0.12-3.49) |
| **Radiotherapy** |  |  |  |  | 0.3650 |  |  |
| No | 3732 | 55 | 21293 | 2.58 (1.98-3.36) |  | 1 (Ref) | 1 (Ref) |
| Yes | 10236 | 166 | 56760 | 2.92 (2.51-3.41) |  | 1.14 (0.84-1.55) | 1.12 (0.82-1.53) |
| **Trastuzumab** |  |  |  |  | 0.2731 |  |  |
| No | 11535 | 180 | 65273 | 2.76 (2.38-3.19) |  | 1 (Ref) | 1 (Ref) |
| Yes | 2433 | 41 | 12780 | 3.21 (2.36-4.36) |  | 1.22 (0.87-1.71) | 1.01 (0.70-1.44) |
| **Age 60-69** |  |  |  |  |  |  |  |
| **Reimbursement type** |  |  |  |  | 0.6944 |  |  |
| National health insurance | 4673 | 174 | 26006 | 6.69 (5.77-7.76) |  | 1 (Ref) | 1 (Ref) |
| Medical aid | 135 | 6 | 756 | 7.94 (3.57-17.68) |  | 1.27 (0.58-2.79) | 1.21 (0.55-2.66) |
| **Chemotherapy(any)** |  |  |  |  | 0.8486 |  |  |
| No | 1778 | 67 | 10104 | 6.63 (5.22-8.43) |  | 1 (Ref) | 1 (Ref) |
| Yes | 3030 | 113 | 16658 | 6.78 (5.64-8.16) |  | 1.03 (0.76-1.39) | 0.98 (0.71-1.36) |
| **Endocrine treatment** |  |  |  |  | 0.3941 |  |  |
| No | 1463 | 55 | 8001 | 6.87 (5.28-8.95) |  | 1 (Ref) | 1 (Ref) |
| Tamoxifen | 651 | 19 | 3747 | 5.07 (3.23-7.95) |  | 0.75 (0.44-1.25) | 0.77 (0.45-1.31) |
| Aromatase inhibitor^b^ | 2694 | 106 | 15014 | 7.06 (5.84-8.54) |  | 1.02 (0.74-1.42) | 1.08 (0.77-1.52) |
| **Ovarian function suppression^c^** | |  |  |  | NA |  |  |
| No | 4808 | 180 | 26762 | 6.73 (5.81-7.78) | NA | NA | NA |
| Yes | 0 | NA | NA | NA | NA | NA | NA |
| **Radiotherapy** |  |  |  |  | 0.2862 |  |  |
| No | 1584 | 68 | 9025 | 7.53 (5.94-9.56) |  | 1 (Ref) | 1 (Ref) |
| Yes | 3224 | 112 | 17736 | 6.31 (5.25-7.60) |  | 0.85 (0.63-1.14) | 0.84 (0.61-1.13) |
| **Trastuzumab** |  |  |  |  | 0.4677 |  |  |
| No | 4121 | 153 | 23154 | 6.61 (5.64-7.74) |  | 1 (Ref) | 1 (Ref) |
| Yes | 687 | 27 | 3608 | 7.48 (5.13-10.91) |  | 1.18 (0.79-1.78) | 1.21 (0.78-1.88) |
| **Age ≥70** |  |  |  |  |  |  |  |
| **Reimbursement type** |  |  |  |  | 0.0317 |  |  |
| National health insurance | 1638 | 116 | 8465 | 13.70 (11.42-16.44) |  | 1 (Ref) | 1 (Ref) |
| Medical aid | 80 | 11 | 412 | 26.68 (14.78-48.18) |  | 2.03 (1.10-3.72) | 1.99 (1.08-3.67) |
| **Chemotherapy(any)** |  |  |  |  | 0.9716 |  |  |
| No | 1205 | 90 | 6269 | 14.36 (11.68-17.65) |  | 1 (Ref) | 1 (Ref) |
| Yes | 513 | 37 | 2608 | 14.19 (10.28-19.58) |  | 1.00 (0.68-1.47) | 0.97 (0.64-1.47) |
| **Endocrine treatment** |  |  |  |  | 0.1303 |  |  |
| No | 511 | 43 | 2483 | 17.32 (12.84-23.35) |  | 1 (Ref) | 1 (Ref) |
| Tamoxifen | 354 | 20 | 1934 | 10.34 (6.67-16.03) |  | 0.59 (0.35-1.00) | 0.60 (0.35-1.03) |
| Aromatase inhibitor^b^ | 853 | 64 | 4460 | 14.35 (11.23-18.33) |  | 0.82 (0.55-1.20) | 0.80 (0.54-1.19) |
| **Ovarian function suppression^c^** | |  |  |  | NA |  |  |
| No | 1718 | 127 | 8877 | 14.31 (12.02-17.02) | NA | NA | NA |
| Yes | 0 | NA | NA | NA | NA | NA | NA |
| **Radiotherapy** |  |  |  |  | 0.7612 |  |  |
| No | 991 | 72 | 5143 | 14.00 (11.11-17.64) |  | 1 (Ref) | 1 (Ref) |
| Yes | 727 | 55 | 3734 | 14.73 (11.31-19.19) |  | 1.06 (0.74-1.50) | 1.06 (0.74-1.52) |
| **Trastuzumab** |  |  |  |  | 0.8427 |  |  |
| No | 1609 | 120 | 8348 | 14.37 (12.02-17.19) |  | 1 (Ref) | 1 (Ref) |
| Yes | 109 | 7 | 529 | 13.23 (6.31-27.75) |  | 0.99 (0.47-2.07) | 0.89 (0.40-1.98) |

NA, not available

^a^ log-rank test

^b^ letrozole, anastrozole, exemestane

^c^ goserelin, leuprolide
